# Culture-Independent Genotyping Improves Surveillance of *Neisseria gonorrhoeae*, Especially in Oropharyngeal Samples, the Netherlands, 2017 to 2018

**DOI:** 10.3390/pathogens11111344

**Published:** 2022-11-14

**Authors:** Michiel H. C. Slaats, Brian M. J. W. van der Veer, Lieke B. van Alphen, Christian J. P. A. Hoebe, Nicole H. T. M. Dukers-Muijrers, Petra F. G. Wolffs

**Affiliations:** 1 Department of Medical Microbiology, Care and Public Health Research Institute (CAPHRI), Maastricht University Medical Center (MUMC+), P.O. Box 5800, 6202 AZ Maastricht, The Netherlands; 2 Department of Sexual Health, Infectious Diseases and Environmental Health, South Limburg Public Health Service, P.O. Box 33, 6400 AA Heerlen, The Netherlands; 3 Department of Health Promotion, Care and Public Health Research Institute (CAPHRI), Maastricht University Medical Center (MUMC+), P.O. Box 5800, 6202 AZ Maastricht, The Netherlands

**Keywords:** *Neisseria*, genotyping, culture-independent, gonorrhoeae, surveillance, culture

## Abstract

It is important i to monitor the transmission and antimicrobial resistance of *Neisseria gonorrhoeae* (NG). Current surveillance relies on culturing, which frequently fails. Previously, a culture-independent genotyping method was developed based on NG multi-antigen sequence typing (NG-MAST). To determine whether crucial sequence types (STs) are missed during culture-dependent surveillance, NG-positive NAAT samples were genotyped, and the results of the culture-positive and culture-negative samples were compared. In total, 196 NG-positive NAAT samples, from January 2017 until August 2018, which were also routinely cultured, were retrospectively included. Genotyping was successful in 152 NAAT samples (77.0%), 33 NAAT samples failed, and 11 NAAT samples showed possible mixed strain infections. Oropharyngeal samples (n = 16) showed the largest increase in typing rate from 6.3% (1/16) success in culture-dependent genotyping to 81.3% (13/16) in culture-independent genotyping. Nine genogroups (n ≥ 5 samples) were found; all included both culture-positive and culture-negative NG. However, culture-independent surveillance revealed 14 additional STs in the culture-negative samples. Overall, culture-dependent surveillance could detect all genogroups, indicating that major trends could be identified with culture-dependent surveillance. However, culture-independent surveillance provides more STs, mixed infections and more oropharyngeal samples, giving a more detailed view and could result in an earlier detection of outbreaks and transmission.

## 1. Introduction

*Neisseria gonorrhoeae* (NG) is one of the most common bacterial sexually transmitted infections (STIs) [1]. Its high incidence and its increasing resistance to antibiotic therapy is a cause for major concern [1,2]. Surveillance of NG is an important public health method to monitor NG transmission and resistance [1,3]. Therefore, multiple surveillance programs have been initiated in Europe to monitor the transmission and antimicrobial resistance of NG [3,4,5]. However, the currently available typing methods used in these surveillance programs rely on culturing [6,7,8]. Relying on culturing has major drawbacks. First, not all countries and laboratories perform culturing for NG. Instead, its diagnosis is based on nucleic acid amplification tests (NAAT). This is in line with current recommendations to use NAAT for the diagnosis of NG infections [9]. Second, when culturing of NG is performed, it frequently fails due to various factors, such as demanding nutritional and environmental growth requirements or commensal flora [9]. These factors resulted in only 37.8% of NG cases being culture-confirmed in the Netherlands in 2020 [10]. As culture-dependent surveillance can only be applied on a subset of all samples, there is a possibility of missing outbreaks in the population and the spread of STs potentially associated with resistance.

Surveillance using culture-independent typing methods could vastly improve current culture-dependent surveillance. By not relying on cultures, genetic surveillance could yield a more complete view of circulating STs due to the increased typing rate compared to culture-dependent surveillance. Additionally, theoretically, molecular techniques could detect some STs that might be more difficult to culture due to specific nutritional needs or because they favour a specific anatomical site, such as the oropharynx, which has lower sensitivity in culturing [11,12,13]. In addition, culture-independent methods could potentially be more suitable for retrospective or longitudinal studies where the loss of culturability during storage could be an issue

Previously, we developed a culture-independent genotyping method based on NG multi-antigen sequence typing (NG-MAST). This method achieves higher typing rates than routine culture-based NG-MAST by eliminating the need for culture [14]. Using this method as a proxy for culture-independent surveillance, we aimed to investigate whether using a culture-independent genotyping method would improve current surveillance. For this, we investigated if the increased typing rate of the culture-independent approach would lead to more STs and genogroups as seen in culture-dependent surveillance. Furthermore, the typing rates per sample material were compared between strategies.

## 2. Results

### 2.1. Culture-Independent Typing of Clinical Samples

In total, 152/196 (77.0%) NG-positive NAAT samples were successfully genotyped using the culture-free NG-MAST method, including two possible mixed infections. Of the typed samples, 3/152 required touchdown PCR to be genotyped. This PCR protocol employs a higher initial annealing temperature that is progressively decreased, resulting in increased sensitivity and yield [15]. The samples that were unsuccessful (e.g., failed) did not yield an amplified product of either *porB* or *tbpB* or had ambiguous base calls in the sequence data that indicated mixed strain infections. Of the 44 samples that failed, 11 samples showed different *porB* or *tbpB* sequences in the same sample, indicating a possible mixed infection. The program used was not able to call the different sequences for the mixed infections. The other 33 samples failed to amplify a product in the initial PCR, possibly due to inhibitory factors in the clinical samples. Failure was related to the Cq value of the original NG NAAT testing as the failed samples showed a median Cq value of 28, and the typed samples showed a median Cq value of 27 (Mann–Whitney U-test, W = 2681.5, n = 196), *p* = 0.046 (Supplementary Figure S1). Possible mixed infections (n = 13), including two that were genotyped successfully, were mainly observed in urine samples (8/13), although they were also seen in all other sample sites (vaginal 1/13, anorectal 3/13, and 1/13 oropharyngeal). All anatomical sites showed good typing rates: urine 79/102 (77.5%), vaginal swab 19/22 (86.4%), anorectal swab 41/56 (73.2%) and oropharyngeal 13/16 (81.3%). These different anatomical sites showed no significant difference in typing rate (χ^2^ (3, n = 196) = 1.71, *p* = 0.63) (Table 1).

### 2.2. Comparison of Surveillance Methods

**Figure 1:**
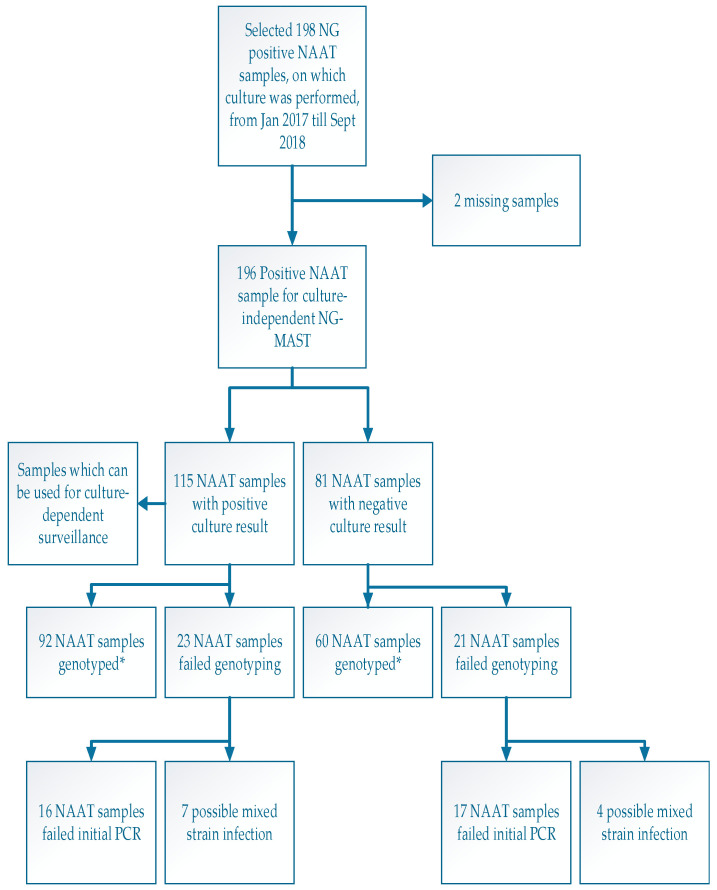
Overview of the study design. Definition of abbreviations: NG = *Neisseria gonorrhoeae*; NAAT = nucleic acid amplification test; NG-MAST = NG multi-antigen sequence typing. * These NAAT samples were successfully genotyped resulting in 152 samples.

To compare the different surveillance methods, genotyping was performed on NG-positive NAAT samples, and a comparison was made between those that were culture-positive and those which were culture-negative. The culture was not repeated, and previous culture results were used as a proxy for typing success. Using culture-independent genotyping, 80.0% (92/115) of the culture-positive samples were successfully genotyped, and 74.1% (60/81) of the culture-negative samples were genotyped; these proportions were similar (χ^2^ (1, n = 196) = 0.96, *p* = 0.33) (Figure 1).

The culture-dependent typing rate of urine samples did not differ compared to the culture-independent method (χ^2^ (1, n = 204) = 0.42, *p* = 0.52). However, as seen in Table 1, a higher typing rate with the culture-independent method was seen for the other anatomical sites: vaginal (χ^2^ (1, n = 24) = 9.82, *p* = 0.002) and anorectal (χ^2^ (1, n = 112) = 4.66, *p* = 0.03). The largest difference was seen for oropharyngeal samples, where using culture-dependent genotyping, only 1 of 16 samples (5.9%) was culture-positive and could be genotyped, while using culture-independent genotyping, 13 of the 16 NAAT samples (81.3%) could be genotyped (χ^2^ (1, n = 32) = 18.3, *p* < 0.001) (Table 1).

Of the 23 failed culture-positive samples, 7 samples showed a mixed infection. A mixed-infection was also seen in 4/21 failed culture-negative samples. The software used in this study was not able to define the alleles in these samples. However, two of the successfully genotyped samples showed a mixed infection (one culture-positive and one culture-negative). The software was able to call an allele in these samples, but only the predominant allele could be called.

### 2.3. Sequence Types and Genogroups

**Figure 2:**
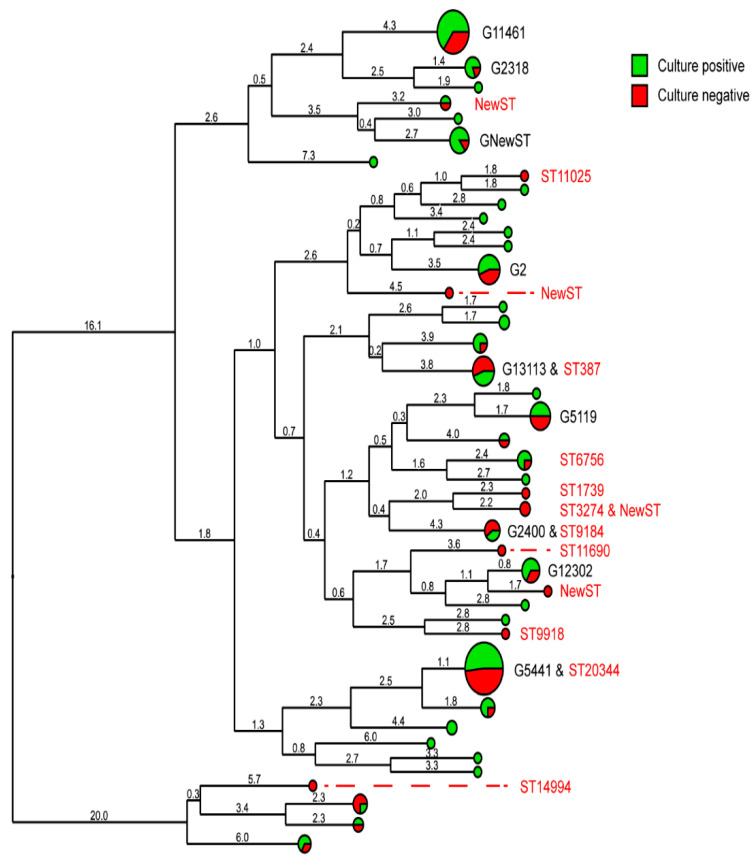
Dendogram of *Neisseria gonorrhoeae* sequence strains made from concatenated sequences (*porB* and *tbpB*) clustered with the unweighted pair group method with the arithmetic mean, the Netherlands, January 2017–September 2018 (n = 152). The sequence type of each sample is shown at the tips of the dendrogram, and the sequence types in red indicate the strains missed in culture-based surveillance. The branch length depicts the percentage of dissimilarity.

In this dataset of 152 genotyped clinical samples, 9 genogroups were identified (Figure 2): G11461 (n = 20, 13 culture-positive and 7 culture-negative), G2318 (n = 5, 4 culture-positive and 1 culture-negative), G New ST (n = 7, 6 culture-positive and 1 culture-negative), G2 (n = 9, 5 culture-positive and 4 culture-negative), G13113 (n = 9, 4 culture-positive and 5 culture-negative), G5119 (n = 8, 4 culture-positive and 4 culture-negative), G2400 (n = 5, 2 culture-positive and 3 culture-negative), G12302 (n = 6, 4 culture-positive and 2 culture-negative) and G5441 (n = 29, 15 culture-positive and 14 culture-negative). All genogroups consisted of both culture-positive and culture-negative samples, although G2318, G13113, G5119, G2400 and G12302 each had fewer than five culture-positive samples. These would not have been identified as genogroups without the culture-negative samples. In total, 66 different STs were identified; in culture-dependent surveillance, 52 STs would have been identified and 14 STs were missed because of negative culture results: New ST1, ST11025, New ST2, ST387, ST6756, ST1739, ST3274, New ST3, ST9184, ST11690, New ST4, ST9918, ST20344 and ST14994 (Figure 2). These STs originated from different anatomical sites: eight urine samples, three anorectal swabs, three vaginal swabs and two oropharyngeal swabs. ST9184 was found in both the vaginal and oropharyngeal sites.

## 3. Discussion

Our data demonstrate that culture-dependent surveillance detects most STs and large genogroups. However, some STs and large proportions of genogroups are missed in culture-dependent surveillance because of negative culture results. Moreover, culture-independent genotyping demonstrated a vastly increased typing rate in vaginal and anorectal samples and especially in oropharyngeal samples. While current culture-dependent surveillance programmes show a good overview of the most common circulating NG strains, culture-independent surveillance adds details and could aid in a better understanding of the transmission of NG strains, especially if oropharyngeal samples are involved.

Culture-independent surveillance was able to detect 14 additional STs that were missed using culture-dependent surveillance. The additional STs were single isolates, except for ST9184 and ST20344 of which two isolates were found. Using only culture-dependent surveillance data, the STs in the genogroups were seen, but some genogroups did not have sufficient culture-positive samples to be defined as genogroups if culture-independent surveillance was not used. The higher typing rate of culture-independent surveillance likely explains the additional STs and the size of the genogroups. In this set of samples, the typing rate increased from 58% (culture-dependent) to 77% (culture-independent). The earlier detection of genogroups and the emergence of (new) STs can be key in the early detection of outbreaks and the management of potential sources.

Mixed strain infections are currently missed using routine culture-dependent surveillance as typically only one colony from the culture is used for genotyping. Culture-independent surveillance showed 13 possible mixed strain infections in our dataset. Previous studies also found mixed infections; however, the importance of these possible mixed strain infections is not yet known [16,17]. Theoretically, these mixed strain infections could have a major role in antimicrobial resistance as resistance genes can be exchanged. The data-analysis program we used was not able to call alleles in our samples with mixed infections, except for two cases (two urine samples) as the loads of the different strains were likely vastly different. However, only the predominant allele was called, and the other allele was ignored. Other molecular techniques, such as metagenomics, might provide a solution to this problem. Future studies are needed to further elucidate the mixed strain infections of NG and define the implications in transmission and surveillance.

Current culture-dependent surveillance is biased towards genital and anorectal samples, as most culture-positive samples were from these anatomical sites, and only a fraction were from the oropharyngeal site, as this site is difficult to culture [11,12]. Our results confirm the low success rate of oropharyngeal culture, as only 1 of the 16 (5.9%) samples submitted for culture was positive and could be genotyped. In contrast, culture-independent genotyping was able to genotype 13 of the 16 NAAT (81.3%) samples. Due to other commensal *Neisseria* species, direct genotyping of oropharyngeal samples is difficult. However, van de Veer et al. [13] demonstrated that the primers used for culture-free NG-MAST are specific for NG and do not cross-react with other commensal *Neisseria* species; therefore, all genotyped oropharyngeal samples are NG. The ability to genotype more NG strains in oropharyngeal samples is very important in surveillance, as these oropharyngeal strains could have a crucial role in the emergence of antimicrobial resistance. This is because NG can obtain resistance genes from commensal *Neisseria* species, commonly present in the oropharynx, via horizontal gene transfer [18].

The variety of STs found in this study may be an underestimation. Only a portion of the NAAT-positive samples are cultured in routine diagnostics. A culture was performed in only 68.3% of all gonorrhea-positive patients in the Netherlands in 2020 (data from the Gonococcal Resistance to Antimicrobials Surveillance (GRAS) programme [9]). Second, if culturing is performed, the culture success rate is generally low. Culture in our subset of samples showed a relatively high success rate of 58% compared to the national average (<40%) [19]. Hence, discrepancies in genogroups and STs may be larger if culture-independent surveillance is performed in a setting with a lower culture success rate [20].

Part of the current surveillance programs include monitoring for the emergence of resistance. While culture-independent surveillance can detect more STs than culture-dependent surveillance, it does not provide antibiotic susceptibility data. Other molecular methods, such as NG-STAR or whole genome sequencing (WGS) can provide insights into antimicrobial resistance. However, these methods should be adapted so they can be performed on clinical samples without culture, including extragenital samples. If feasible, this approach could be the cornerstone of future surveillance programs. Nevertheless, culture remains crucial, as mutations do not always result in phenotypic resistance, and the effect of new mutations should still be addressed in surveillance using culture.

In conclusion, no major differences in STs between culture-dependent and culture-independent surveillance were observed. However, because culture of NG-positive samples is not always performed or frequently fails, especially in samples with a low bacterial load such as oropharyngeal samples, the current culture-dependent surveillance strategy misses STs, genogroups and mixed infections, which could be crucial in the early detection of outbreaks. Furthermore, culture-independent genotyping increases the typing rate of samples other than urine and vastly improves the typing rate of oropharyngeal samples. Hence, the implementation of culture-independent methods will provide a more detailed view of the circulating NG strains in the population. This could aid in further understanding and prevention of NG transmission.

## 4. Materials and Methods

### 4.1. Clinical Samples

We retrospectively included all NG-positive NAAT samples (n = 198) of patients, regardless of Cq value, who were also submitted to routine culture for NG between January 2017 and September 2018 from our STI clinic (South Limburg Public Health Service). Two samples were missing, resulting in 196 NG positive NAAT samples, consisting of 102 male urine samples, 22 vaginal swabs, 56 anorectal swabs and 16 oropharyngeal swabs. After the initial NAAT test (Roche Cobas 4800, Woerden, The Netherlands) 1 mL of urine or COBAS medium was stored at −20 °C until genotyping. Original NG NAAT testing was not repeated after thawing of samples because the Cobas medium used retains viable DNA well over prolonged storage [21].

Routine culture was successful in 115/196 samples, while 81/196 samples were culture-negative. All samples were taken at the STI clinic South Limburg, and either urine or sterile swabs were collected. The sterile cotton swabs were placed in Stuart medium (Thermo Fisher, Bleiswijk, The Netherlands) for transport. Samples were transported to the laboratory daily at room temperature. These samples were inoculated on GC lect agar (Beckton Dickinson, Vianen, the Netherlands) and were placed in a CO^2^ incubator (5% CO^2^) at 37 °C for 48 h. Plates were checked daily for growth, and suspected colonies were identified using MALDI-TOF (Biomerieux, Amersfoort, the Netherlands).

### 4.2. Culture-Independent Genotyping

Culture-free NG-MAST was used as the culture-independent genotyping method in this study and was performed according to the specifications of van der Veer et al. [14]. Additionally, all samples that failed the first genotyping attempt (n = 36) were repeated using touchdown PCR to increase the sensitivity [15]. If they failed the touchdown PCR, the NAAT samples were defined as a genotyping failure. Afterwards the same precipitation and Sanger sequencing method was used as described by van der Veer et al. [14].

### 4.3. Data Analysis

As cultures were performed as part of the routine diagnostic procedures, these were not repeated in this study to avoid bias due to freeze–thaw cycles. To compare the culture-dependent and independent surveillance, we assumed that the previously positive cultures would have a typing rate of 100%. Furthermore, we assumed that the ST found with culture-free NG MAST would match with the culture because the genetic material in the samples remained the same, independent of the method, as van der Veer et al. demonstrated in a small set of paired samples [14].

Bionumerics (version 7.6, Applied Maths, Sint-Martens-Latem, Belgium) was used to edit and trim the Sanger sequence data as previously described [14]. The online curated NG-MAST database was used to call alleles and STs. If sequence data were not sufficient to determine an allele because ambiguity and multiple different *porB* or *tbpB* sequences were present, the sample was defined as mixed infection. However, samples were regarded as successfully genotyped if one different allele was more prominent, leading to allele calling by Bionumerics. Afterwards, a phylogenetic tree was constructed of the concatenated *porB* and *tbpB* sequences using multiple sequence alignment and the unweighted pair group method with the arithmetic mean. We defined genogroups as having ≥5 samples, which had 1 identical allele, while the other was at least 99% identical, as this grouping correlates to closely related strains and leads to more robust analysis [8,22]. Using the phylogenetic tree, genogroups and STs were compared between the culture-positive and culture-negative samples. Chi-square tests were used to analyse differences between the culture-positive and culture-negative groups. The Mann–Whitney U-test was used to compare Cq-values between typed and failed samples. Statistical analysis was performed using SPSS 25 (IBM, Armonk, NY, USA).

## Figures and Tables

**Table 1:** Culture-free genotyping of *Neisseria gonorrhoeae* of culture-positive and culture-negative samples per sample material, including a comparison between culture-based and culture-free genotyping, the Netherlands, January 2017–September 2018 (n = 196).

	Culture-Positive			Culture-Negative			Typing-Rate Culture-Independent			Typing-Rate Culture-Dependent *	
**Sample material**	Typed	Failed	Total	Typed	Failed	Total	Culture positive	Culture negative	Total	Total	*p*-value **
**Urine (n = 102)**	60 (2 mixed)	15 (4 mixed)	75	19	8 (2 mixed)	27	80.0%	70.4%	77.5%	73.5% (75/102)	0.63
**Vaginal swab (n = 22)**	8	1 (1 mixed)	9	11	2	13	88.9%	84.6%	86.4%	40.9% (9/22)	0.002
**Anorectal swab (n = 56)**	23	7 (2 mixed)	30	18	8 (1 mixed)	26	76.7%	69.2%	73.2%	53.6% (30/56)	0.03
**Oropharyngeal swab (n = 16)**	1	0	1	12	3 (1 mixed)	15	100.0%	80.0%	81.3%	6.3% (1/16)	<0.001

* Culture positivity was used as proxy for genotyping success; ** Chi-square tests were used to analyse the differences.

## Data Availability

The data presented in this study are available on request.

## Supplementary Materials

The following supporting information can be downloaded at: https://www.mdpi.com/article/10.3390/pathogens11111344/s1, Figure S1: Overview of the Cq-values of the original NAAT of the typed and failed samples.
