# Supplementary material for: Culture-Independent Genotyping Improves Surveillance of Neisseria gonorrhoeae, Especially in Oropharyngeal Samples, the Netherlands, 2017 to 2018
Source: Pathogens. 2022 Nov 14;11(11):1344. doi: 10.3390/pathogens11111344 (PMC9697099; doi:10.3390/pathogens11111344)
Supplement: Supplementary file 1 [file pathogens-11-01344-s001.zip › pathogens-1837681-supplementary.pdf]

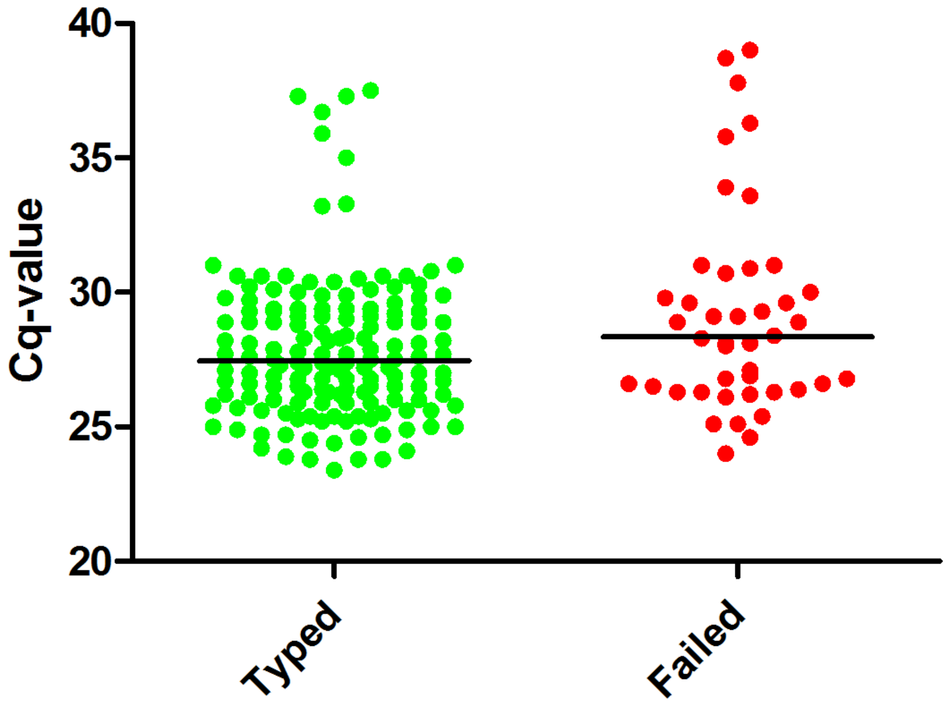

Supplementairy Figure S1. Overview of the Cq-values of the original NAAT of the typed and failed samples. Median Cq value of the typed samples is 27 and 28 of the failed samples, p = 0.046 (Mann-Whitney U-test (W = 2681.5, N = 196).
